# Hinokinin, an Emerging Bioactive Lignan

**DOI:** 10.3390/molecules190914862

**Published:** 2014-09-17

**Authors:** Maria Carla Marcotullio, Azzurra Pelosi, Massimo Curini

**Affiliations:** Department of Pharmaceutical Sciences, University of Perugia, via del Liceo 1, 06123 Perugia, Italy; E-Mails: azzurra.pelosi@gmail.com (A.P.); massimo.curini@unipg.it (M.C.)

**Keywords:** cubebinolide, cytotoxicity, *Trypanosoma*, Chagas disease, antigenotoxic activity

## Abstract

Hinokinin is a lignan isolated from several plant species that has been recently investigated in order to establish its biological activities. So far, its cytotoxicity, its anti-inflammatory and antimicrobial activities have been studied. Particularly interesting is its notable anti-trypanosomal activity.

## 1. Introduction

**Figure 1:**
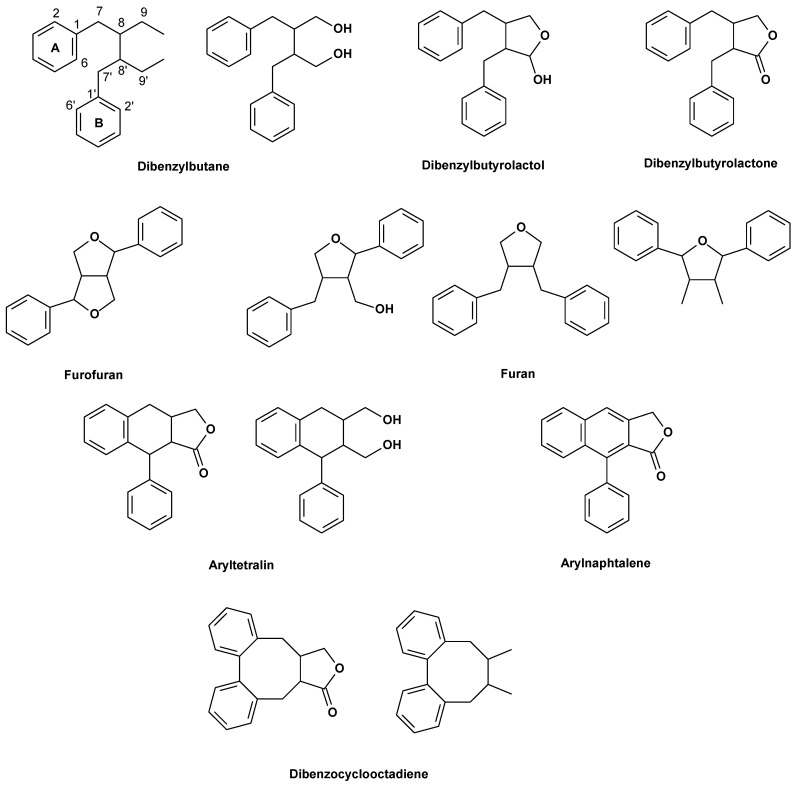
General classes of lignans.

Lignans are important components of foods and medicines biosynthetically deriving from the radical coupling of two molecules of coniferyl alcohol at C-8/C-8′ positions (Figure 1). They are classified in different groups—dibenzylfuran, dihydroxybenzylbutane, dibenzylbutyrolactol, dibenzylbutyrolactone, aryltetraline lactone and arylnaphtalene derivatives—on the basis of the skeleton oxidation [1] and of the way in which oxygen is incorporated into the skeleton [2] (Figure 1). Podophyllotoxin and deoxypodophyllotoxin are, perhaps, the most important biologically active lignans, and their properties have been broadly reviewed [3,4].

In these last years, the biological activities of several lignans have been studied in depth [5,6,7] and among them hinokinin (**1**) is emerging as a new interesting compound. The aim of this review is to examine hinokinin (**1**) from a phytochemical and biological point of view. Peer-reviewed articles on hinokinin were acquired via the Scopus, SciFinder, and PubMed databases.

## 2. Phytochemistry

**Scheme 1:**
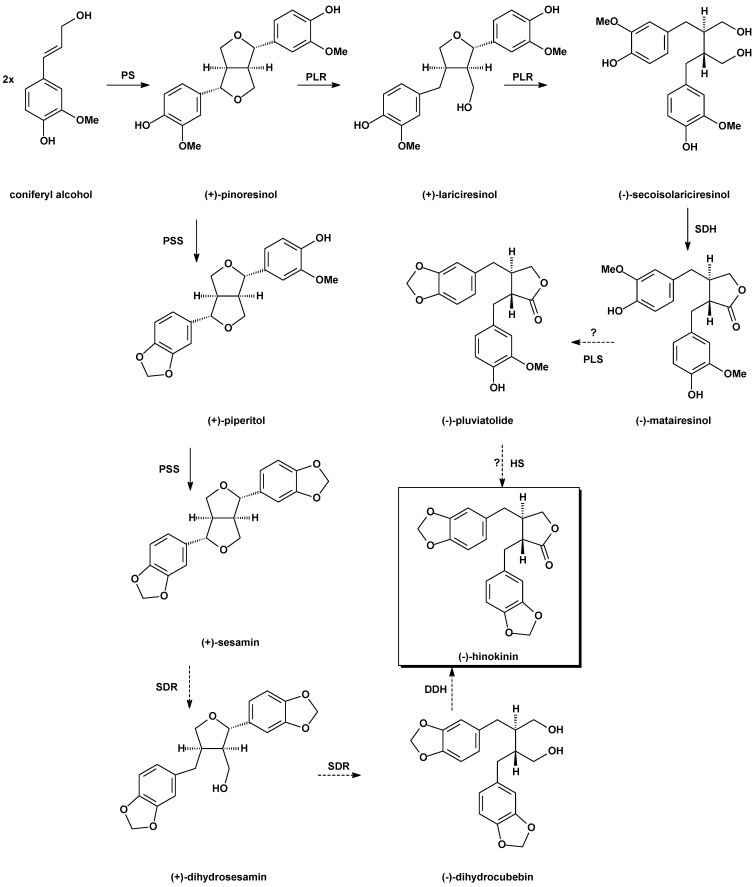
Proposed biosynthetic pathways for hinokinin (**1**). PS, pinoresinol synthase; PLR, pinoresino-lariciresinol reductase; SDH, secoisolariciresinol dehydrogenase; PLS, pluviatolide synthase; HS, hinokinin synthase; PSS, piperitol-sesamin synthase; SDR, sesamin-dihydrosesamin synthase; DDH, dihydrocubebin dehydrogenase [13].

**Figure 2:**
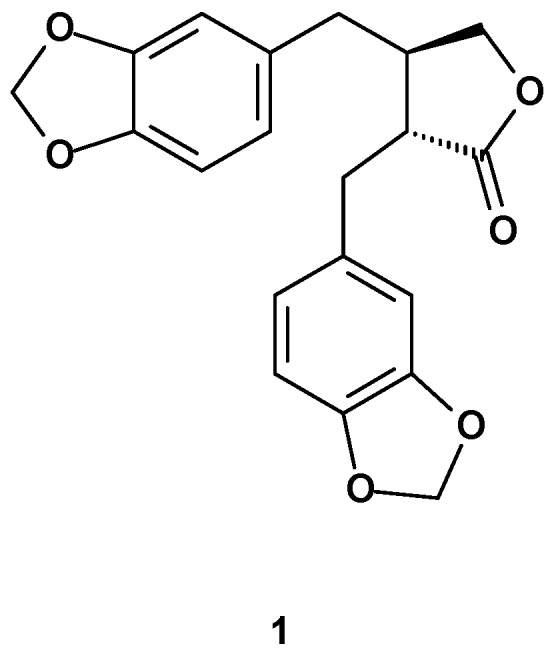
Hinokinin’s structure.

Hinokinin (**1**, Figure 2) was isolated for the first time by Yoshiki and Ishiguro in 1933 from the ether extract of hinoki wood (*Chamecyparis obtusa* Sieb. et Zucc.) as a colorless crystalline compound [8] and later Mameli, Briggs and Keimatsu established the identity of hinokinin with cubebinolide [9,10,11]. Haworth and Woodcock determined the *trans* configuration of the lactone ring by synthesis [12]. Biosynthesis of (−)-hinokinin was recently studied in *Linum corymbulosum* Reichenb by Bayindir *et al.* [13]. Starting from the observation that callus cultures of *L. corymbulosum* accumulate **1** [14], and according to the lignan composition found in *Chamaecyparis obtusa* by Takaku [15], the authors proposed two different pathways for the biosynthesis of hinokinin starting from (+)-pinoresinol (Scheme 1).

In the first pathway, pinoresinol is reduced to secoisolariciresinol by a pinoresinol-lariciresinol reductase (PLR-Lc1), followed by the formation of the methylenedioxy bridges. In the second pathway, there is the formation of the methylenedioxy bridges on pinoresinol to give sesamin and the latter is then converted into dihydrocubebin and hinokinin. By the isolation of (PLR-Lc1), the enzyme responsible of the enantiospecific conversion of (+)-pinoresinol to (−)-secoisolariciresinol, they established that the first pathway is operative in hinokinin biosynthesis.

## 3. Distribution

After the first isolation from *C. obtusa*, hinokinin was isolated from *C. formonensis* [16] and from several other plants [17,18,19,20,21,22,23], such as for example *Zanthoxylum simulans* [24], *Z. naranjillo* [25], *Z. lemairie* [26], *Z. monophyllum* [27], *Z. pistaciiflorum* [28], *Z. ailanthoides* [29]. It was also found in different species of *Phyllanthus* [30,31,32], *Aristolochia* [33,34,35,36,37,38,39,40,41,42,43], *Piper* [44,45,46,47,48,49,50,51,52,53], *Virola* [54,55,56,57,58], *Linum* [59,60,61,62,63]. Another genus that produces hinokinin is *Bursera*. Compound **1** was found in *B. cuneata* and *B. citronella* by Koulman [64] and in *B. simaruba* by Maldini *et al.* [65].

## 4. Biological acitivities

### 4.1. Cytotoxic Activity

Hinokinin (**1**) was found to be a component of several cytotoxic extracts such as the petroleum ether fraction of a 75% ethanol extract of *Zanthoxylum ailanthoides* Sieb. & Zucc. stem bark [66]. The cytotoxicity of hinokinin (**1**) has been investigated by several authors against different cancer lines: P-388 (murine lymphocytic leukemia), HT-29 (human colon adenocarcinoma), A-549 (human lung adenocarcinoma) and MCF-7 (human breast adenocarcinoma) [18,67].

Ikeda *et al.* tested hinokinin (**1**) isolated from *Anthriscus sylvestris* [68] against B16F10 (murine metastatic melanoma), HeLa (human cervical cancer) and MK-1 (murine gastric adenocarcinoma) cell lines using the 3-[4,5-dimethylthiazol-2-yl]-2,5 diphenyltetrazolium bromide (MTT)-microculture assay [69]. Results are reported in Table 1.

Hinokinin was also examined as antitumor promoter agent in a human cytomegalovirus (HCMV) immediate early (IE) antigen expression in human lung adenocarcinoma (A-549) cells [70]. After establishing the ID_50_ (dose causing 50% inhibition) in A-549 cell line (40.72 μg/mL), the authors found that **1** was able to reduce the IE antigen expression in HCMV-infected lung cancer cells in a dose-dependent manner (ID_1_: 81, ID_10_: 57% reduction, respectively).

**Table 1:** Cytotoxicity of hinokinin (**1**) against selected tumor cell lines ^a^.

	P-388	HT-29	A-549	MCF-7	B16F10	HeLa	MK-1
Hinokinin (**1**)	1.54 ^b^	4.61 ^b^	8.01 ^b^		2.72 ^c^	2.58 ^c^	1.67 ^c^
		11.4 ^d^	26.1 ^d^	13.8 ^d^			
	5.87 ^e^	3.52 ^e^	6.61 ^e^				
Mithramycin	0.08 ^b^	0.07 ^b^	0.06 ^b^				
	0.06 ^e^	0.08 ^e^	0.07 ^e^				
Adriamycin		0.1 ^d^	0.02 ^d^	0.1 ^c^			
Podophyllotoxin ^e^					0.001	0.0025	0.006

^a^ ED_50_ μg/mL; ^b^ [18]; ^c^ [68]; ^d^ [67]; ^e^ [28].

According to Suffness and Pezzuto pure compounds are considered to have antitumor activity if they show ED_50_ values less than 4 μg/mL [71]. From this point of view, hinokinin (**1**) could be regarded as an antitumoral compound against P-388, HT-29, B16F10, HeLa and MK-1 cell lines. Mansoor *et al.* evaluated the apoptosis induction of hinokinin in human hepatoma HuH-7 cells [72]. Hinokinin significantly reduced viability of HuH-7 cells and it showed to be a strong inducer of apoptosis, inducing 2.4- and 2.5-fold increases in apoptotic cells as compared to controls. Furthermore, hinokinin was found to be highly toxic using the brine shrimp letality test (BST) [73].

Recently Awale *et al.* studied the cytotoxicity of several lignans isolated from *W. indica*, against Panc-1 cancer cell line (human pancreatic cancer) [74]. They found that (8S,8′S)-(+)-hinokinin as well as other lignans, such as (+)-arctigenin, with the same stereochemistry, were inactive against Panc-1 cell line, whereas the (−) enantiomers were cytotoxic. These results indicate that the absolute configuration of (−)-enantiomers is required for the cytotoxicity. Hinokinin resulted ineffective against HONE-1 (nasopharyngeal carcinoma) and UGC-3 (gastric adenocarcinoma) cell lines [75].

### 4.2. Anti-Inflammatory Activity

It is well known that inflammation is a key event in cancer development [76] and for this reason nowadays the anti-inflammatory activity of natural compounds is broadly studied. Hinokinin (**1**) was shown to be a potent inhibitory compound on human neutrophil superoxide generation and elastase release by neutrophils with an IC_50_ of 0.06 ± 0.12 μg/mL and an inhibitory percentage of 24.7 ± 6.2 at 10 μg/mL, respectively (diphenyleneiodonium: IC_50_ 0.54 ± 0.21, phenylmethylsulfonyl fluoride: 35.24 ± 5.62% of inhibition) [77].

Furthermore, it was able to inhibit LPS-induced nitric oxide generation in RAW264.7 macrophages (IC_50_ 21.56 ± 1.19 μM; aminoguanidine: 6.51 ± 1.15 μM) [78]. da Silva *et al.* studied the *in vivo* anti-inflammatory activity of hinokinin in the rat paw oedema reduction assay. Hinokinin (**1**) was shown to possess a good anti-oedema activity (in terms of efficacy) in a dose dependent manner (at the dose of 30 mg/kg it induced 63% of reduction, similar to indomethacin at the dose of 5 μg/mL) [79]. This anti-inflammatory activity was accompanied by an analgesic effect as demonstrated by the same authors in the acetic acid-induced writhing test in mice. Compound **1** produced high inhibition levels of the algogenic process (97%).

Immunosuppressive activity can play an important role in managing and resolving inflammation. Regarding the immunosuppressive activity of hinokinin (**1**), it has no activity against NFAT transcription factor [80], but it was found active in the lipopolysaccharide (LPS) induced cytokine production assay for IL-10, IL-12, and TNF-α [81] and remarkably active in a lymphocyte transformation assay [82] (Table 2). Recently, Desal *et al.* studied the anti-inflammatory effects of hinokinin against IL-6 and TNF-α, establishing that **1** exerts its anti-inflammatory effects via an NFκB-dependent mechanism [83].

**Table 2:** Immunosuppressive activity of hinokinin (**1**).

	Cytokine Production Ratio ^a^				LTI ^d^
	TNF-α	IL-12	IL-10	IL-6^c^	
Hinokinin (**1**)	0.36 ^b^	0.44 ^b^	0.37 ^b^		25.94 ± 1.02
	77.5 ^c^			20.5	
LPS ^b^	1	1	1		
Prednisolone ^b^	0.6	0.2	0.41		
Dexamethasone					9.17 ± 0.53

^a^ Cytokine production ratios were expressed as ratios to cytokine production induced by LPS; ^b^ Hinokinin tested at 10 μg/mL, Prednisolone tested at 0.3 μg/mL [81]; ^c^ IC_50_ values are given in μg/mL [83]; ^d^ Lymphocyte transformation inhibition, IC_50_ given in μg/mL [82].

Lima *et al.* evaluated the anti-inflammatory and analgesic activities of bark crude dichloromethane extract (BCED) of *Z. riedelianum* [84]. They found that BCED was able to reduce carrageenan-induced rat paw oedema after 4 h at the dose 100 mg/Kg (% inhibition: 57.4; indometacin 43.2% at 10 mg/Kg). One of the components of the active extract was hinokinin. The authors suggested that the extract could display anti-inflammatory activity associated with COX inhibition. Moreover, BCED displayed a central analgesic activity too.

### 4.3. Anti-Parasitic Activities

#### 4.3.1. Activity against *Trypanosoma cruzi*

Hinokinin (**1**) showed an interesting activity against *Trypanosoma cruzi*, the responsible of Chagas’ disease, a neglected protozoan disease that affects some 8 million people in Latin America [85,86]. Currently, there are only two effective drugs for Chagas’ disease treatment, namely nifurtimox and benznidazole (BZN), which both cause serious side effects, therefore, there is an urgent demand for the discovery of safer and more effective new therapeutic compounds. *T. cruzi* has a complex life cycle characterized by several developmental forms present in vertebrate and invertebrate hosts. This parasite exists in at least three morphologically distinct forms: infective (metacyclic or blood trypomastigotes), insect borne (epimastigotes) which replicate in the vector, and intracellular replicative (amastigotes) [87]. Hinokinin (**1**) in these last years has been studied as an interesting antitripanosomal compound [86]. In 2005 de Souza *et al.* testedhinokinin (**1**) *in vitro* against free amastigotes forms of Y strain of *T. cruzi* [88]. They found that **1** had an IC_50_ of 0.7 μM compared to BZN (IC_50_ 0.8 μM) (Table 3).

**Table 3:** *In vitro* anti-trypanosomal activity of hinokinin (**1**).^a^

	Free Amastigotes Y Strain ^b^	Intracellular Amastigotes CL Strain ^c^	Epimastigotes Forms of CL Strain ^c^	% of Parasitaemia Reduction ^c^	Trypomastigotes ^d^	Intracellular Amastigotes ^d^
Hinokinin (**1**)	0.7	18.36	0.67	70.8	94.49	>141.24
BZN	0.8	20.00	30.89	29.0	146.02	>190.83

^a^ IC_50_ (μM); ^b^ [89]; ^c^ [90]; ^d^ [90].

In view of its anti-trypanosomal activity, hinokinin (**1**) was later selected to be assayed against epimastigote and intracellular amastigote forms of *T. cruzi*, both *in vitro* and *in vivo* assays [91] (see Table 3). In the *in vivo* assays obtained results showed that the treatment with hinokinin (**1**)promoted 70.8% of parasitaemia reduction in the parasitaemic peak, while benznidazole displayed approximately 29.0% of parasite reduction.

The antitrypanosomal activity of hinokinin was determined using the MTT assay by Sartorelli and coworkers [90]. They evaluated **1** against trypomastigotes and intracellular amastigotes of *T. cruzi*. Results are shown in Table 3. In order to study the toxicity of hinokinin (**1**) in mammalian cells, Sartorelli also studied hinokinin’s hemolytic activity and cytotoxicity. Hinokinin was shown to be effective on trypomastigotes, but it resulted toxic to mammalian cells and with a low parasite selectivity (selectivity index <1) [90].

To obtain better efficacy of this promising lead compound towards the intracellular forms of the parasite, Saraiva *et al.* prepared and investigated the effect of a new formulation using biodegradable polymers, such as poly(D,L-lactide-co-glycolic acid; PLGA), for the controlled release of hinokinin. The treatment of infected mice with hinokinin-loaded microparticles was able to provoke significant decrease in parasitemia levels compared with those observed in untreated controls [91]. Furthermore, Saraiva *et al.* showed that the administration of hinokinin-loaded microparticles was able to reduce the number of parasites more than hinokinin itself, in the course of the overall infection.

The reduction of tissue parasitism upon treatment with hinokinin (**1**), was evaluated *in vivo* by Esperandim and coworkers by quantifying the enzyme β-galactosidase expressed by the CLB5 clone strain of *T. cruzi* [92,93]. Treatment of mice infected with *T. cruzi* CLB5 with hinokinin (**1**) promoted significant reduction of tissue parasitism (liver, spleen and heart) compared with data recorded for untreated controls. Treatment with hinokinin (**1**) or benznidazole at a drug concentration of 50 mg/Kg a day, furnished a parasitism reduction of 50.5% or 41.7% in the liver; 71% or 16% in the spleen; and 41.4%, or 30.4% in the heart, respectively. The authors noted that there were some differences between the oral and intraperitoneal administration routes, being the former more effective for all evaluated organs, while BZN administered intraperitonealy was more effective for spleen and heart parasitism reduction [92]. Later, Esperandim evaluated in detail the *in vivo* therapeutic properties of oral administered hinokinin (**1**) against CLB5 strain of *T. cruzi* [93]. Hinokinin was assayed at concentration of 20 and 50 mg/kg. The authors observed that hinokinin at 20 mg/kg reduced the number of circulating forms at peak parasitemia of 51%, while at 50 mg/kg of 34.2%. The karyometry analysis once again showed a better behavior of 20 mg/kg dose (Table 4).

The non-linear behavior between the two doses, with the 20 mg/Kg dose being more effective than the other, has been explained by an immunomodulatory response that hinokinin (**1**) can exert. It is well known that the immunosuppression of chronically infected patients can lead to disease reactivation, with high parasitemia and it has been already reported that hinokinin (**1**) can act as an immunosuppressive compound (see above).

**Table 4:** Karyometry analysis. Mean values of the nuclear area from cells of the spleen, liver, and heart of control groups and mice inoculated with the CL Brener clone strain of *Trypanosoma cruzi* B5.

Groups	Area (μm^2^)		
	Spleen	Heart	Liver
CINF ^a^	10.86 ± 2.45	18.20 ± 8.81	32.99 ± 7.78
C ^b^	8.12 ± 2.04	15.05 ± 8.64	28.56 ± 5.69
Hinokinin 20 ^c^	9.32 ± 2.22	17.48 ± 8.53	30.15 ± 7.90
Hinokinin 50 ^d^	10.00 ± 2.68	18.56 ± 7.74	30.50 ± 7.49
BZN 20 ^c^	9.69 ± 2.50	17.59 ± 7.08	29.46 ± 8.03
BZN 50 ^d^	9.62 ± 2.37	20.42 ± 10.75	28.56 ± 6.45

^a^ CINF: infected not treated animals; ^b^ C: control, uninfected animals; ^c^ Tested dose: 20 mg/kg; ^d^ Tested dose: 50 mg/kg.

#### 4.3.2. Antiplasmodial Activity

Hinokinin was tested for its antiplasmodial activity against 3D7-chloroquine sensitive and Dd2-chloroquine resistant strains of *Plasmodium falciparum*. The IC_50_ of hinokinin (90.7 ± 1.4 μg/mL and 54.4 ± 8.5 μg/mL, respectively; chloroquine IC_50_ 0.094 μg/mL) showed that **1** doesn’t possess significant antimalarial activity against either strain [94].

### 4.4. Antimicrobial Activity

Hinokinin (**1**) has been studied for its bioactivity against several other microorganisms. For example, Silva *et al.* examined the activity of this compound against oral pathogens such as *Enterococcus faecalis*, *Candida albicans* and several *Streptococcus* strains (see Table 5). It can be pointed out from data reported in Table 5 that, although chlorhexidine is much more active than hinokinin, **1** nevertheless showed a discrete antimicrobial activity [95]. Considering this antibacterial activity of hinokinin, Silva *et al.* evaluated the anti-mycobacterial activity of **1** and others lignans [96]. Hinokinin showed to be moderately active against *M. tuberculosis*, with a MIC value equal to 62.5 μg/mL and inactive against *M. kansasii* and *M. avium* (MIC 2000 μg/mL and 500 μg/mL, respectively).

**Table 5:** Minimum inhibitory concentrations (MIC; mM) of hinokinin against oral pathogens.

	*E. faecalis*	*S. salivarius*	*S. sanguinis*	*S. mitis*	*S. mutans*	*S. sobrinus*	*C. albicans*
Hinokinin (**1**)	0.38	0.25	0.25	0.25	0.32	0.28	0.28 ^a^
Chlorhexidine ^b^	5.9	1.7	3.9	5.9	5.9	1.5	7.9

^a^ Fungicidal concentration; ^b^ MIC: μM.

### 4.5. Antiviral Activity

Several research groups studied the antiviral properties of hinokinin against human hepatitis B virus (HBV) [97], human immunodeficiency virus (HIV) [29], SARS-virus (SARS-CoV) [98], and in all cases **1** showed significant antiviral activity.

### 4.6. Genotoxic and Antigenotoxic Activities

In light of the interesting biological activities of hinokinin (**1**) and its potential use as therapeutic agent, it is important to investigate its mutagenic and genotoxic activities. Recently Resende *et al.* used the Ames and Comet assays, to assess the safety of using hinokinin as a drug [99]. In the Comet assay, on Chinese hamster lung fibroblasts (V79), hinokinin was shown to not be genotoxic. In the treatments with hinokinin associated with the known mutagen doxorubicin (DXR), the lower concentrations of **1** (0.5; 1.0 and 2.0 μM) significantly reduce DXR-induced DNA damage. The reduction in the DNA damage frequency ranged from 60.8% to 76.0% and it is not dose dependent.

Resende also showed that hinokinin has a protective effect in preventing clastogenic damage caused by methyl methanesulfonate (MMS), with the percent reduction ranging from 37.4% to 57.6% [100]. Mutagenic activity was evaluated by the Ames test, using the *Salmonella typhimurium* tester strains TA98, TA100, TA97a and TA102, using five different concentrations of hinokinin (9.75–78.0 μg/plate) selected on the basis of a preliminary toxicity test. The mutagenicity assays show that **1** did not induce any increase in the number of revertant colonies relative to the negative control, indicating the absence of any mutagenic activity.

Medola and coworkers studied the mutagenic and/or antimutagenic effects of hinokinin (**1**) *in vivo* using the rat peripheral blood micronucleus test. The differences of micronucleated cells between treated animals and control were not significative, demonstrating no genotoxic effect, while co-exposition of the animals to hinokinin and DXR showed a significant reduction in the frequencies of MNPCEs (micronucleated polychromatic erythrocytes). However, this protective effect of hinokinin was not dose dependent [101].

### 4.7. Target-Based Studies

Hinokinin (**1**) was tested for several other biological activities, such as antispasmodic effect on electrically induced (ECI), acetylcholine induced (AChI) and histamine induced contractions in isolated guinea-pig ileum, using the Ca^2+^ channel blocker verapamil as a positive control [102]. Hinokinin (**1**) significantly inhibited ECI and AChI contractions.

Neurite outgrowth-promoting activity in PC12 cells of hinokinin (**1**) isolated from *C. obtusa* in the presence or absence of Nerve Growth Factor (NGF, 2 ng/mL) was studied [103]. Hinokinin showed potent neurite outgrowth-promoting activities: 76.0% ± 6.0% at 10 μg/mL, and 50.9% ± 2.6% at 5 μg/mL when cultured with NGF, and 33.2% ± 5.4% at 10 μg/mL and 16.5% ± 2.6% at 5 μg/mL without NGF.

Nowadays, it is well established that neurons and glia development is regulated by neurotransmitters. Monoamine neurotransmitters such as dopamine, norepinephrine and serotonine have a positive action as classical growth factors, while glutamate and GABA (γ-aminobutyric acid) are down-regulating proliferation agents [104]. Hinokinin (**1**) showed neuroprotective activity against glutamate induced neurotoxicity in primary cultures of rat cortical cells (at 1.0 μM percentage of protection 42.6 ± 2.4, at 10.0 μM 56.9 ± 3.4; dizocipline maleate, a non-competitive antagonist of NMDA (*N*-methyl-d-aspartate) receptor (one of the glutamate receptors) showed at 1.0 μM 71.7 ± 1.2 and at 10.0 μM 77.4 ± 2.1 percentage of protection) [105]. Furthermore, Timple *et al.* demonstrated that hinokinin is a selective inhibitor of human dopamine and norepinephrine transporters in a noncompetitive manner with a low affinity for the serotonine transporter [106].

Cytochrome P450 (CYP) enzymes play an important role in phase I oxidation metabolism of a widw range of xenobiotics. In humans, 57 isoforms of CYP were identified, CYP3A4, CYP1A2, CYP2A6, CYP2D6, CYP2C8 and CYP2E1 among others.

Methylenedioxyphenyl compounds were well known to inhibit cytochrome P (CYP) reaction because they form stable complexes with CYP enzymes [107]. For this reason, several natural compounds incorporating this structural feature have been studied for their inhibitory activity of CYP enzymes. Hinokinin (**1**) containing two methylenedioxyphenyl rings in the molecules, showed potent CYP inhibition [108]. Later Usia *et al.* showed that hinokinin is active on CYP3A4 but not on CYP2D6 [109] and that CYP3A4 is inhibited in a time-, concentration- and NADPH-dependent manners via the formation of a metabolite intermediate complex [110], therefore, attention should be paid to a probable drug-drug interaction between hinokinin-containing preparations and molecules that are substrates of CYP3A4.

## 5. Conclusions

Lignans represent an important biologically active class of secondary metabolites. The most studied biological activities of these compounds are their antioxidant and anticancer properties. However, in recent years the importance of such metabolites, especially hinokinin, as potential antichagasic agents has been pointed out. In addition, hinokinin was shown to be non-genotoxic and to possess a neuroprotective effects. For all these reasons, hinokinin is emerging as a promising compound with broad and interesting biological activity.
